# Size-, Shape-, and Number Concentration-Dependent Nanoplastics Accumulation and Growth Responses in Lettuce

**DOI:** 10.3390/polym18121436

**Published:** 2026-06-09

**Authors:** Hisayuki Nakatani, Taito Miyaji, Masaki Sakamoto, Suguru Motokucho, Anh Thi Ngoc Dao

**Affiliations:** 1 Chemistry and Materials Engineering Program, Graduate School of Integrated Science and Technology, Nagasaki University, 1-14 Bunkyo-machi, Nagasaki 852-8521, Japananh.dao@nagasaki-u.ac.jp (A.T.N.D.); 2 Organization for Marine Science and Technology, Nagasaki University, 1-14 Bunkyo-machi, Nagasaki 852-8521, Japan

**Keywords:** nanoplastics, plant interaction, particle shape effects, low-density polyethylene, polystyrene

## Abstract

Understanding the ecological impacts of nanoplastics requires evaluation metrics beyond conventional mass-based concentrations. In this study, we investigated the generation, characterization, and phytotoxic effects of environmentally relevant plastic particles in hydroponically grown lettuce (*Lactuca sativa*), focusing on particle size, shape, and number concentration. Low-density polyethylene (LDPE) was degraded using an advanced oxidation process, demonstrating that substantial oxidative degradation is required for the formation of nanoplastics; the resulting LDPE particles exhibited a broad size distribution ranging from the nanoscale to the micrometer scale, containing nanoscale domains (peak size ~20 nm, average size ~30 nm), and showed predominantly ellipsoidal morphologies derived from cross-linked polymer regions. In contrast, polystyrene (PS) particles of defined sizes (~600 nm and ~2000 nm) were prepared via mechanical fragmentation, exhibiting sharp-edged, flake-like morphologies. Laser scanning microscopy revealed uptake and translocation of LDPE particles from roots to aerial tissues, whereas larger PS particles showed limited transport. Growth inhibition analysis based on particle number concentration (10^10^–10^17^ particles/mL) showed a stepwise concentration–response relationship for LDPE particles, with inhibition levels increasing from approximately ~30% at low concentrations to high levels of inhibition at the highest concentrations In contrast, PS particles caused significant root damage (e.g., clear surface disruption observed in microscopy) and growth inhibition (~30–40%) even at relatively low number concentrations (~10^10^–10^12^ particles/mL), likely due to their sharp-edged morphology. Overall, plant responses to plastic particles were governed by an interplay of size, shape, and number concentration, highlighting the importance of particle morphology and concentration metrics in agroecosystem risk assessment.

## 1. Introduction

Plastic pollution has become a widespread environmental issue [1,2,3,4,5,6,7,8,9,10], and microplastics (MP) and nanoplastics (NP) are now recognized as pollutants that are widely distributed in aquatic, terrestrial and atmospheric ecosystems [5,11,12,13,14,15]. The MPs and NPs are generated either when larger plastic waste breaks down due to degradation or are released directly from industrial products and consumer goods, eventually entering the soil and freshwater systems that support agricultural production. Consequently, there is growing attention on the interactions between plastic particles and plants, particularly regarding absorption, translocation, and potential impacts on plant growth and development [16,17,18,19,20]. These studies have demonstrated that, under specific conditions, plastic particles are absorbed by plant roots and transported to above-ground tissues, raising concerns about their potential entry into the food chain [16,17,18]. It has been shown that the extent of uptake by plants strongly depends on particle size, with nanometer-sized particles generally exhibiting higher permeability and mobility than micrometer-sized particles [16]. Consequently, much of the research to date has focused on particle size as a key determinant of plant–plastic interactions. However, plastic particles in the environment exhibit significant heterogeneity not only in size but also in shape, surface properties, and degree of degradation, all of which may influence biological behavior [20].

Furthermore, although MPs and NPs are solids and thus fundamentally different from dissolved chemicals, most studies on plant exposure have used mass-based concentrations to assess phytotoxic effects. However, for particulate pollutants, especially at the nanoscale, mass-based metrics may not adequately represent exposure, because the number of particles increases dramatically as particle size decreases. As a result, the same mass concentration can correspond to vastly different particle numbers depending on particle size. When investigating the effects of particulate pollutants, particle number concentration and particle morphology may therefore serve as more appropriate indicators of exposure than mass concentration alone, as biological interactions are likely governed by particle number, surface area, and contact frequency rather than total mass. Particle number concentration, defined as the number of particles per unit volume (e.g., particles/mL), directly reflects the frequency of interactions between particles and plant tissues. In practice, it can be estimated by combining particle size information with total particle mass under simplified geometric assumptions. Although such estimates involve uncertainties due to heterogeneity and aggregation, they provide a useful approximation of exposure conditions in nanoscale systems. Therefore, systematic studies that quantitatively link particle number concentration to plant growth inhibition remain limited, highlighting the need for further investigation. This is primarily due to the difficulty in preparing environmentally relevant NPs, as their formation requires advanced degradation processes such as advanced oxidation processes (AOP), and sufficiently high levels of oxidative degradation to generate nanoscale particles. In addition, the resulting particles typically exhibit broad size distributions and complex morphologies, making controlled preparation challenging. In this context, the carbonyl index (CI), defined as the ratio of the absorbance of carbonyl groups (typically around 1715 cm^−1^) to that of a reference peak (e.g., 1450 cm^−1^), is widely used as an indicator of oxidative degradation in polymers. As oxidation progresses, the formation of carbonyl-containing functional groups increases, leading to a higher CI value. Therefore, CI provides a useful measure for evaluating the extent of polymer degradation, which is closely related to the fragmentation into micro- and nanoplastics.

Low-density polyethylene (LDPE) and polystyrene (PS) are two of the most abundant plastic polymers detected in the environment [17,18], yet they differ significantly in chemical structure, degradation behavior, and mechanical properties. While both LDPE and PS are susceptible to oxidative degradation and may generate MP and NP, differences in particle shape arise depending on the glass transition temperature and the presence or absence of cross-linked structures. Such differences in particle morphology are considered to be a physical factor independent of particle size and concentration that may significantly influence plant responses. Lettuce (*Lactuca sativa*) was selected as a model plant in this study due to its widespread use in studies of micro- and nanoplastics uptake and phytotoxicity. In addition, lettuce is a commonly consumed leafy vegetable, making it highly relevant for assessing the potential transfer of plastic particles into the human food chain. Furthermore, its suitability for hydroponic cultivation allows controlled exposure conditions. In this study, environmentally relevant particles were prepared from LDPE via advanced oxidation processes (AOP), while PS particles of defined sizes were produced by mechanical fragmentation and size selection. Their uptake, translocation, and growth-inhibitory effects were systematically investigated in hydroponically grown lettuce. To achieve this, we combined visualization techniques with quantitative growth inhibition analysis based on particle number concentration to elucidate how particle size, shape, and concentration interact to govern plant responses to plastic particles. In this study, we employed a laser scanning microscopy approach combined with staining using iDye Poly Pink to visualize the distribution of plastic particles within lettuce tissues. This approach enables direct observation of particle localization in roots, hypocotyls, and cotyledons and provides qualitative insights into uptake pathways and transport behavior. It is also essential for linking particle distribution with biological responses. These results provide new insights into the mechanisms underlying plant–nanoplastic interactions and highlight the importance of considering particle morphology and number concentration in environmental risk assessments.

## 2. Materials and Methods

### 2.1. Materials

LDPE was obtained from Sigma-Aldrich Co. LLC (St. Louis, MI, USA), and its melt index (190 °C/2.16 kg) value was 25 g/10 min. Wako Pure Chemical Industries (Osaka, Japan) provided potassium persulfate (K_2_S_2_O_8_) and PS pellets (the weight-average molecular weight and molecular weight distribution were 3.5 × 10^5^ and 2.1, respectively). Artificial seawater (GEX artificial seawater, GEX Co., Ltd., Tokyo, Japan. https://www.amazon.co.jp/-/en/GEX-Gex-Seawater-6-5-Pieces/dp/B09R9X4Y43?th=1&utm, accessed on 21 April 2026), prepared according to the manufacturer’s instructions, was used as the seawater medium.

### 2.2. Advanced Oxidation Process (AOP) Using Sulfate Ion Radicals in Seawater

The test specimens for LDPE and PS films were ca. 30 mm × 30 mm × 0.050 mm. The films were obtained by compression molding at 180 °C under 10 MPa for 11 min, and were degraded in seawater using a sulfate ion radical in an accelerated degradation method [21]. The procedure was in accordance with our previous reports [21]. (1) Several pieces of each film were put into a 100 mL glass vessel containing 20 mL of seawater solution with 0.54 g K_2_S_2_O_8_ at ca 65 °C for 12 h under stirring with a stirrer tip speed of ca 100 rpm. (2) An equal amount of K_2_S_2_O_8_ seawater solution was added to compensate for the consumption of the oxidant, and its degradation was carried out for 12 h under the same conditions. (3) The five pieces of the film were then transferred to a new 100 mL glass vessel containing 20 mL of seawater solution with 0.54 g K_2_S_2_O_8_, and the degradations were started again under the same conditions. The enhanced degradation method was run for a given number of days using (1) to (3) as one set. The pH value of the solution was changed from 8.2 to 3 during each set (the pH of the seawater was initially 8.2, and the SO_4_•^−^ was gradually converted to SO_4_^2−^, reducing the pH of the seawater solution to 3 at the time of the daily exchange [21]). The degraded films were used as “degraded LDPE and PS”.

The degradation procedure using AOP was performed according to our previously reported method [21], in which the formation and fragmentation behavior of environmentally relevant micro- and nanoplastics from LDPE and PS were systematically investigated. In particular, this method has been demonstrated to produce plastic particles with broad size distributions ranging from the nanoscale to the micrometer scale under environmentally relevant degradation conditions [21].

### 2.3. Fragmentation Methods

After the seawater degradation was performed using AOP degradation, the degraded LDPE fragmentation was obtained by detaching the degraded surfaces using vibration from a vortex mixer (vortexer) purchased from Heathrow Scientific^®^ LLC (Vernon Hills, IL, USA). The two sheets of degraded film (1 × 1 cm) were put into a conical tube (φ30 × 115 mm) with 5 mL pure water, and then the conical tube was then shaken using the vortex mixer at a speed of 3000 rpm for 1 h. On the other hand, particle sizes of PS samples were not governed by degradation time but by subsequent mechanical processing and filtration. Therefore, the degraded PS film was ground into fine particles using a mortar, and PS particles of the required particle size were obtained through preliminary and final filtration.

### 2.4. Methods for Calculating the Particle Number Concentrations of NP and MP Samples

An aqueous solution of degraded LDPE particles, which had been converted into nanoparticles using a vortex mixer, was centrifuged at 80,000 rpm using an ultracentrifuge (Optima TLX: Beckman Coulter, Inc., Brea, CA, USA) to separate the NP [LDPE particles with nanoscale features (~20 nm, peak size; average ~30 nm)] particles. After vacuum drying, the total weight of the particles was measured. In the case of NP from degraded PS, after pulverization, the PS (600 nm) was pre-filtered using a membrane filter (hydrophilic PTFE type, Advantec Toyo Kaisha, Ltd., Tokyo, Japan) with a pore size of 1 μm, and then filtered using a membrane filter with a pore size of 0.2 μm. On the other hand, the MP-type PS (2000 nm) was obtained by pre-filtering through a membrane filter with a pore size of 3 μm, followed by filtration through a membrane filter with a pore size of 1 μm. After filtration, the filter paper was immersed in a glass bottle containing pure water and subjected to ultrasonic treatment for 5 min; the resulting aqueous solution was then ultracentrifuged to obtain PS (600 nm) and PS (2000 nm) particles, respectively. These were then dried under reduced pressure, and their total weights were measured. Some of these particles were destroyed during the ultracentrifugation process, and since the particles remaining after centrifugation had agglomerated, only a portion of them returned to their original size when dispersed in water. Therefore, four samples were prepared per batch. For three of these samples, the weight was measured using the method described above, and the average weight of the three samples was calculated. The remaining sample was not subjected to ultracentrifugation and was used in the experiment as is. Particle size was measured using this sample that had not undergone centrifugation. Particles were characterized using dynamic light scattering (DLS) to obtain size distributions in aqueous suspension, while their morphology was examined by SEM observations. For PS particles, size fractions were controlled by mechanical fragmentation followed by membrane filtration with defined pore sizes. The particle number concentration was calculated using the average weight of three samples, following the procedure described below. After measuring the particle size (longest dimension) of two types of NP and one type of MP, we calculated the volume per particle based on their respective shapes, and then multiplied that volume by the density to determine the mass per particle. The number of particles was calculated by dividing the total mass by the mass per particle, and by dispersing these particles in a specified volume of pure water, we obtained the desired particle number concentration (particles/mL). It should be noted that the calculated particle number concentrations are based on assumed particle geometries and average sizes, and do not explicitly account for aggregation, partial fragmentation, or deviations from idealized shapes. This calculation is based on fundamental physical relationships between particle size, density, and mass, and does not rely on a previously established empirical method. Therefore, the calculated particle number concentrations should be interpreted as approximate values representing the order of magnitude of particle abundance. It should also be noted that, unlike engineered nanomaterials, environmentally generated plastic particles are inherently heterogeneous in size and morphology, and therefore the use of well-defined standard particles does not necessarily represent realistic environmental conditions.

### 2.5. Characterization and Analysis

Scanning electron microscopy (SEM) analysis was conducted using a JSM-7500FAM (JEOL, Tokyo, Japan) at an electron beam voltage of 5.0 kV. The working distance was approximately 3 × 4 mm. The samples were placed in a drying oven maintained at 27 °C for 30 min and sputter-coated with gold before undergoing SEM imaging.

The hydrodynamic size was determined under pure water suspension at 20 °C using an ELSZ-2000ZS dynamic light scattering (DLS) analyzer manufactured by Otsuka Electronics (Osaka, Japan). The hydrodynamic size of the fragment contained in the conical tube after processing was determined by measuring the path length and width of a borosilicate glass standard fluorescence cell (10 mm each) and the capacity (3.5 mL). The DLS analyzer was used to measure the particle size distribution. The range of sizes was measured from 0.6 nm to ca. 15 µm, with the DLS analyzer operating at a temperature of 20 °C and using pure water (viscosity 0.8878 cP, refractive index 1.3328) as the solvent for each measurement. The DLS analyzer performed 25 accumulations for each sample.

The transform infrared spectra of 16 scans were measured with a Fourier transform infrared spectrometer Jasco FT-IR 660 plus (Jasco, Tokyo, Japan) with a resolution of 4 cm^−1^ over the entire mid-IR range (400–4000 cm^−1^). The carbonyl index (CI), defined as the ratio of the absorbance of carbonyl groups (around 1715 cm^−1^) to a reference peak (e.g., 1450 cm^−1^), was used as an indicator of oxidative degradation, as commonly reported in previous studies [21].

Due to the formation of highly cross-linked and insoluble structures during AOP degradation of LDPE, conventional molecular weight analysis using size exclusion chromatography (SEC) was not feasible.

### 2.6. Hydroponic Growth of Lettuce

The hydroponic cultivation conditions were similar to those reported in previous studies [16,17]. To evaluate the effects of nanoplastics (NP) and microplastics (MP) derived from degraded LDPE and PS on the early growth of lettuce, a hydroponic cultivation experiment was conducted. For cultivation, plastic cases with internal dimensions of 3 cm × 3 cm × 1 cm were used, and phenolic foam sponges cut to 2.5 cm × 2.5 cm × 1.0 cm (purchased via Amazon, supplier information not available, Tokyo, Japan. https://www.amazon.co.jp/dp/B0F7KFK3MS, accessed on 21 April 2026) were placed inside the cases. A hole approximately 1.5 cm in diameter was made in the center of the sponge, and two seeds of Early Sunny Lettuce (purchased via Amazon, supplier information not available, Tokyo, Japan. https://www.amazon.co.jp/dp/B0F5GJZLGB, accessed on 21 April 2026) were sown.

The treatment groups were set as follows: (1) distilled water (control), (2) water containing LDPE particles with nanoscale features (~20 nm), (3) water containing PS (600 nm), and (4) water containing PS (2000 nm). On the day of sowing (Day 0), 3 mL of each treatment solution was administered, and thereafter, 1 mL was added to each pot daily for 8 days from Day 1 to Day 8. Watering was performed uniformly at 2:00 PM each day.

The cultivation environment was maintained at a room temperature of 20 °C, and plants were exposed to plant growth lights (manufacturer unknown, Japan. 21 μmol m^−2^s^−1^) daily from 10:00 AM to 5:00 PM. Outside of the illumination period, the entire plastic case was covered with aluminum foil to ensure complete darkness. To prevent sedimentation, the NP solution was gently stirred immediately before use to ensure uniformity, and the same procedure was applied to all treatment groups. Cultivation was conducted under these conditions for 9 days, and germination status, plant height, number of leaves, and any visual abnormalities were recorded daily. Where necessary, traits such as fresh weight and root length were measured on the final day.

The cultivation was conducted under these conditions for 9 days, as preliminary experiments confirmed that this period allowed stable and reproducible evaluation of early growth responses. Germination status, plant height, number of leaves, and visual abnormalities were recorded daily. Where necessary, traits such as fresh weight and root length were measured on the final day. This cultivation period is also consistent with commonly reported early-stage growth assays in plant toxicity studies.

Growth inhibition (%) was calculated using the following equation:

Inhibition (%) = [(Control − Treatment)/Control] × 100

where “Control” and “Treatment” represent the measured values of each growth parameter (e.g., root length, fresh weight, or plant height) in the control and particle-exposed groups, respectively.

### 2.7. Method for Observing the Distribution of Fine Particles Within Lettuce Using Staining

To evaluate the distribution of NP and MP within lettuce, a laser microscope observation was carried out with a digital microscope (Keyence VHX-8000, Keyence, Osaka, Japan) using iDye Poly Pink (Jacquard Products, Tokyo, Japan. https://www.amazon.co.jp/dp/B00C1JVYHG?ref=nav_ya_signin, accessed on 21 April 2026) staining. Lettuce cotyledons cultured for 9 days in water containing stained NP or MP particles were cut lengthwise from root to hypocotyl with a knife, and the cross-sections were observed under a laser microscope. The remaining parts of the cotyledons were observed without being cut. This staining approach was developed in the present study as a practical method to visualize plastic particle distribution within plant tissues, since standardized methods for such visualization are currently limited.

### 2.8. FT-IR Analysis of Dyed LDPE Nanoplastics in Lettuce Roots

For FT-IR analysis, root samples of lettuce exposed to nanoplastics were carefully collected, lightly rinsed with pure water, and dried at room temperature. The samples were then directly analyzed using FT-IR spectroscopy without further chemical treatment.

## 3. Results and Discussion

### 3.1. Characterization of Particles

**Figure 1:**
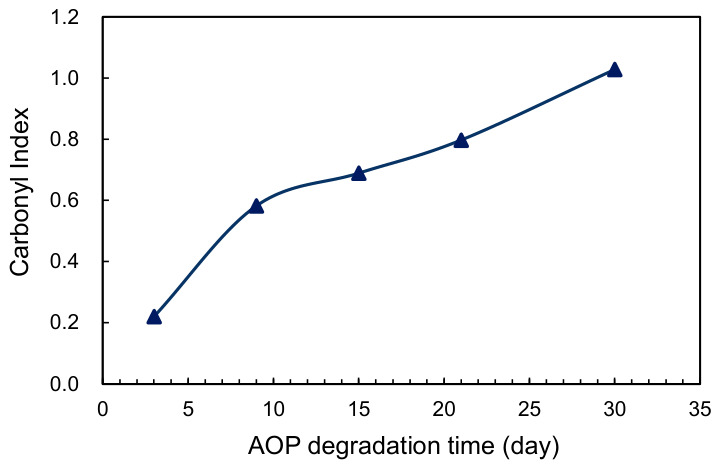
Change in carbonyl index for AOP degradation time of degraded LDPE.

**Figure 2:**
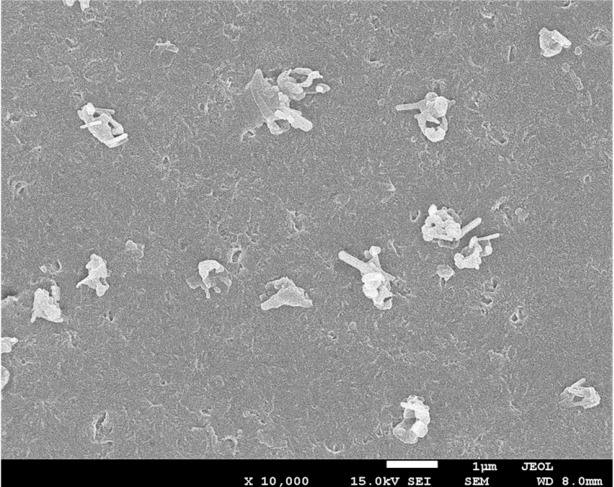
SEM image of LDPE particles detached from the surface after 24 days of AOP degradation: This condition was selected to clearly visualize particle morphology, particularly the rounded and ellipsoidal shapes derived from cross-linked regions, which are less distinct at earlier degradation stages. The particles exhibit rounded and ellipsoidal shapes without sharp edges.

**Figure 3:**
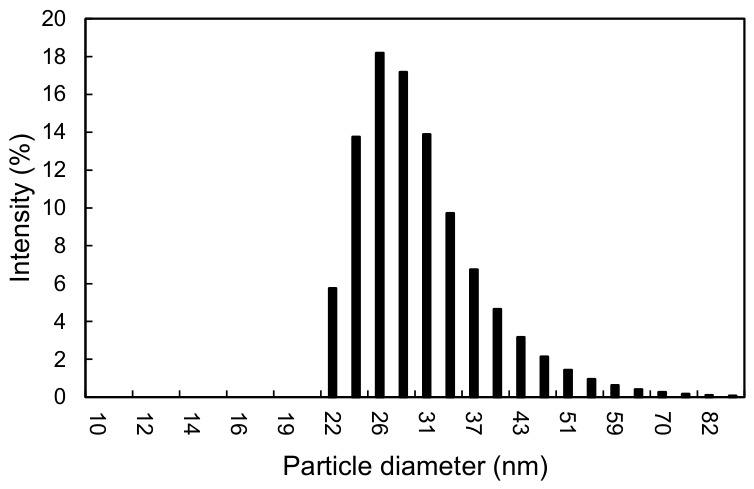
Particle size distribution of LDPE particles obtained after 15 days of AOP degradation: This condition was selected as a representative and reproducible state for particle preparation used in subsequent experiments.

It should be noted that the LDPE particles obtained in this study exhibit a broad size distribution, ranging from the nanoscale to the micrometer scale, based on DLS analysis, while SEM provided complementary morphological information. While the ecological impact of nanoplastics (NPs) is largely determined by their size and quantity, the degree of degradation and particle shape should also be considered. Figure 1 shows the temporal change in the carbonyl index (CI = *I*_1715 cm_^−1^/*I*_1450 cm_^−1^) of degraded LDPE. Environmentally relevant NPs are clearly generated through degradation, and a relatively high degree of degradation is required for spontaneous NP formation. In particular, LDPE, which exhibits high flexibility at room temperature, must undergo severe degradation in order to be fragmented into fine particles such as NP. As shown in Figure 1, the CI was low, at approximately 0.2, 3 days after the start of degradation, but rose to 0.6 by 9 days. This trend indicates that a degradation period longer than approximately 9 days was required to obtain a sufficient amount of nanoscale particles for reliable characterization under the present experimental conditions. Figure 2 shows the SEM image of LDPE particles detached from the surface after 24 days of AOP degradation. These particles are characterized by smooth, rounded edges without sharp facets. Different degradation times were used for morphological observation and particle size analysis. The SEM image in Figure 2 corresponds to a longer degradation period (24 days), which was selected to clearly highlight particle morphology, particularly the rounded and ellipsoidal features. In contrast, the particle size distribution shown in Figure 3 corresponds to 15 days of degradation, which was selected as a practical and representative condition for particle preparation used in subsequent experiments. Thus, these figures serve complementary purposes in illustrating particle characteristics at different stages of degradation. Similar morphological trends were also observed at 15 days, although less distinctly. As shown in Figure 2, the shapes of LDPE particles were not flaky, as is often seen in plastic that has delaminated due to degradation [21], but rather, many specimens exhibited elliptical shapes with rounded edges, similar to those observed in partially melted cross-linked rubber samples. Since cross-linking reactions occur during the radical polymerization process, it is known that LDPE contains cross-linked regions [21,22]. These cross-linked regions do not melt completely even when heated and, like ultra-high-molecular-weight polyethylene, remain as gel-like unmelted portions [22]. Cross-linking can freeze an instantaneous anisotropic chain conformation, leading to residual anisotropic stress and direction-dependent contraction. Under such conditions, non-spherical shapes, such as ellipsoidal morphologies, may emerge as mechanically stable configurations resulting from a balance between entropic elasticity and interfacial energy. In our previous study [21], we demonstrated that during the AOP degradation, LDPE selectively sheds these cross-linked regions to form NPs. Based on these findings, we assumed that NPs produced from the degraded LDPE are elliptical in shape. These nanoscale features should not be interpreted as crystalline lamellae; rather, they originate from cross-linked network domains formed during oxidative degradation. Therefore, the degradation process was evaluated based on carbonyl index and fragmentation behavior rather than molecular weight reduction.

**Figure 4:**
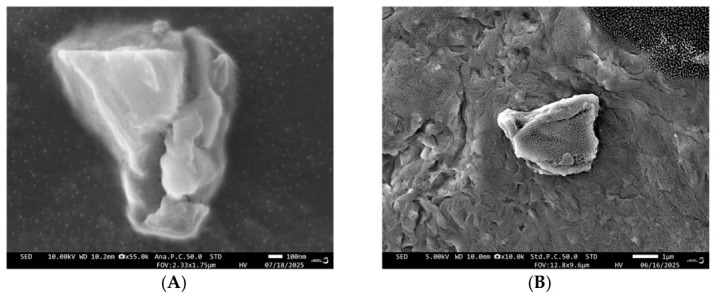
SEM images of AOP-degraded PS particles for 15 days: (**A**) Nano-sized PS [PS (600 nm)], (**B**) Micro-sized PS [PS (2000 nm)]. The particles show flake-like morphologies with relatively sharp edges and angular features.

Simon et al. calculated the ratio of the short axis to the long axis (L) of MP particles (*n* = 398) and found it to be 0.67 ± 0.22 [23]. Assuming that the ratio of the particle thickness to the short axis is constant, the thickness was estimated to be 67% of the short axis. Based on these findings, assuming an ellipsoidal shape and the density (ρ) of the material, the particle weight was calculated from the particle volume using the following equation: a particle weight = π × 0.67^3^ × L^3^/6 × ρ. We investigated the optimal AOP degradation time (9 days or longer) that yielded a relatively uniform particle size distribution and sufficient yield. As shown in Figure 3, setting the AOP degradation time to 15 days resulted in NPs with a relatively narrow particle size distribution showing a nanoscale-dominated distribution with a peak around ~20 nm, while larger particles are also present. It should be noted that the size distribution shown in Figure 3 was obtained from DLS measurements and is independent of the SEM observations presented in Figure 2. The average particle size was approximately 30 nm due to the presence of larger particles in the distribution. A broad size distribution, ranging from the nanoscale to the micrometer scale, was confirmed based on DLS analysis, while SEM provided complementary morphological information. Although TEM analysis would provide higher-resolution visualization of nanoscale particles, the present study focuses on environmentally relevant heterogeneous particle systems; therefore, DLS was used to evaluate particle size distributions in aqueous conditions. After 15 days of AOP degradation, we named the NPs obtained from AOP degradation “LDPE particles with nanoscale features (~20 nm)” and used them in an experiment in which they were added to hydroponically grown lettuce. On the other hand, the AOP degradation behavior of PS differs significantly from that of LDPE. At an AOP degradation time of 15 days, the CI value was approximately 0.35, which is about half of that observed for LDPE. LDPE is produced via radical polymerization under high temperature and pressure [24,25], and is known to contain cross-linked regions that may influence oxidative behavior. The CI value is presented here as a representative indicator of oxidative degradation, rather than as a parameter for detailed quantitative comparison. Consequently, some of the polymer chains contain oxygen at the time of polymerization; in other words, they are oxidized (degraded) [24,25]. Furthermore, since PS tends to decompose into low-molecular-weight compounds when subjected to severe degradation [26], it is expected that recovery of the NP will be difficult. Taking into account the degradation behavior of PS described above, although the CI value is approximately half that of LDPE, the AOP degradation time was set to 15 days, the same as for LDPE. Since degraded PS does not easily fragment into fine particles in water [26], it was ground into fine particles using a mortar. Since the particle size distribution of the degraded PS particles obtained by this method was broad, we narrowed the distribution to the required range by performing pre-filtration. Unlike LDPE, PS does not exhibit a clear time-dependent evolution toward nanoscale particles during AOP degradation. Instead, particle formation required mechanical fragmentation, and thus particle size was controlled by post-processing rather than degradation time. Figure 4 shows SEM images of the degraded PS particles used in this study: PS (600 nm) and PS (2000 nm). The corresponding particle size distributions are shown in Figure S1. In contrast, PS particles exhibit angular, flake-like shapes with distinct sharp edges, which is clearly different from the morphology of LDPE particles. Depending on the presence or absence of cross-linking regions and differences in fragmentation methods, the shapes of these degraded PS particles differed significantly from those of degraded LDPE particles. As shown in Figure 4, PS (600 nm) and PS (2000 nm) were ground at room temperature below the glass transition temperature of PS, resulting in a flake-like morphology with relatively sharp edges. This flake-like morphology is typical of MP recovered from the ocean [27]. Cózar et al. have proposed the formula V = 0.1 × L^3^—where L is the major axis length—multiplied by a shape factor α = 0.1, as a method for calculating the volume of flake-shaped microplastics, which are the predominant form in the marine environment [28]. The weight per particle of PS (600 nm) and PS (2000 nm) was calculated by multiplying the volume equation proposed by Cózar by the density of PS, and this value was used to determine the particle number concentration.

### 3.2. Growth-Inhibitory Effects of LDPE Particles on Hydroponic Lettuce Growing

**Figure 5:**
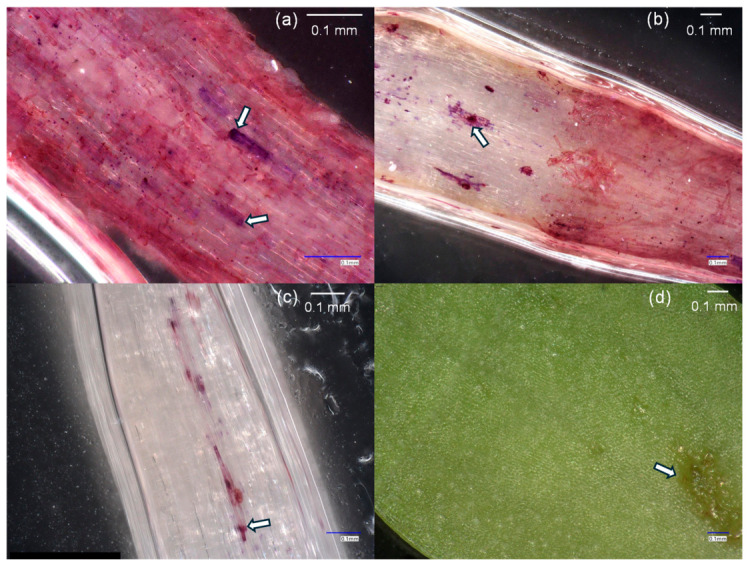
Laser scanning microscopy images showing the uptake and tissue-specific distribution of iDye Poly Pink-stained LDPE particles with nanoscale features (~20 nm) nanoparticles (4.83 × 10^15^ particles/mL) in hydroponically grown lettuce seedlings. (**a**) Root, (**b**) Lower hypocotyl, (**c**) Upper hypocotyl, and (**d**) Cotyledon. Panels (**a**–**c**) show hand-sectioned cross-sections. Arrows indicate the locations of LDPE nanoparticle accumulation. Signals observed in the cotyledons are relatively weak and localized, appearing as faint fluorescence near the vascular regions.

Although multiple growth parameters, including germination, plant height, leaf development, and root growth, were monitored, root-related measurements showed the most consistent and sensitive response and were therefore selected for detailed analysis. LDPE particles caused growth inhibition in hydroponically grown lettuce, particularly affecting root development. This inhibition is likely associated with the accumulation and aggregation of nanoscale particles within root tissues, which may interfere with water and nutrient uptake. Figure 5 shows laser scanning microscopy images illustrating the uptake and tissue-specific distribution of iDye Poly Pink-stained LDPE particles (~20 nm) in hydroponically grown lettuce seedlings. No detectable fluorescence signal was observed in samples without particle exposure under the same imaging conditions, indicating minimal background staining. LDPE particles caused growth inhibition in hydroponically grown lettuce, particularly affecting root development. This inhibition is likely associated with the accumulation and aggregation of nanoscale particles within root tissues, which may interfere with water and nutrient uptake, as supported by the observations in Figure 5. The aggregation of stained LDPE particles observed in cross-sections of roots, the lower hypocotyl, upper hypocotyl, and cotyledons decreased significantly from the roots toward the upper parts, indicating that nanoparticle migration is restricted. As is evident from the photograph in Figure 5a, the entire root is stained with iDye Poly Pink, indicating that numerous LDPE NPs have penetrated the root cells and are aggregating inside them. The size of plastic particles has a significant impact on their uptake by roots. Larger particles are less likely to adhere to the cell walls of plants, which are rich in cellulose, and therefore are less likely to penetrate into root cells. As particle size decreases, the likelihood of plastic particles penetrating into plants increases [29,30,31].

**Figure 6:**
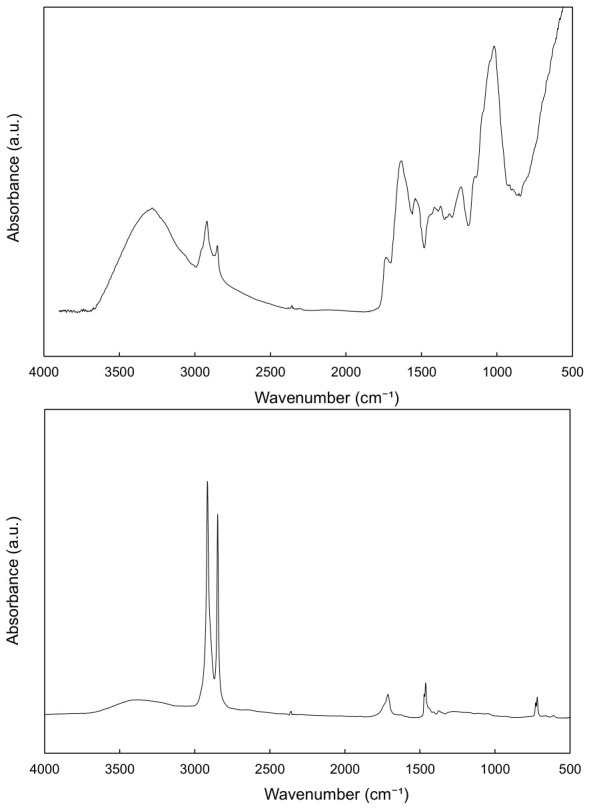
FT-IR spectra of roots of hydroponically grown lettuce after exposure to LDPE particles with nanoscale features (~20 nm) nanoparticles (4.83 × 10^16^ particles/mL) (**top**), and an LDPE sample degraded by AOP for 15 days (**bottom**).

The LDPE particles with nanoscale features (~20 nm) nanoparticles used in this study exhibited a negative zeta potential of −16 ± 10 mV, likely due to degradation. This zeta potential measurement indicates the presence of negative functional groups on the particle surface. Generally, plant roots are composed of cell walls consisting mainly of pectin. Therefore, during hydroponic cultivation, the root surface carries a negative charge due to the ionization of pectin carboxyl groups, although this is pH-dependent. However, in this rhizosphere, under conditions where multivalent cations such as calcium ions are present, the electric double layer breaks down, so it appears that repulsive forces based on negative charges do not occur. In fact, as shown by the IR peaks in Figure 6, sharp double peaks at 2920 and 2850 cm^−1^—presumed to originate from LDPE—are observed on the root surface. The reference spectrum corresponds to LDPE degraded by AOP for 15 days. Although CH_2_ stretching vibrations are also present in natural plant lipids, the sharp double peaks observed at approximately 2920 and 2850 cm^−1^ in roots exposed to LDPE particles are highly consistent with those of untreated LDPE and cannot be explained by biological components alone. This spectral feature differs from plant-derived components such as cellulose, which exhibit broader and less resolved absorption in the same region. This IR spectrum strongly suggests that LDPE particles are adsorbed or accumulated on the root surface and/or in the apoplastic space. The small size of LDPE particles (~20 nm) significantly contributes to their uptake and transport by roots. Generally, larger particles are less likely to adhere to the cell walls of plants, which are rich in cellulose, and thus are less likely to penetrate into root cells. In other words, as mentioned above, the smaller the particle size, the greater the likelihood of penetration into the root [29,30,31].

Many researchers have reported that while PS plastics with a particle size of 1 μm can penetrate plant tissues, the extent of this penetration is far less than that of nanoscale particles, and that larger plastics are more difficult to transport within plants [30,31]. Sun et al. reported that negatively charged nanoplastics (PS-COOH) were detected more frequently in the apoplast and xylem than positively charged nanoparticles (PS-NH_2_), and they hypothesized that this was because changes in the culture medium and root exudates caused the positively charged PS-NH_2_ to aggregate into larger particles [32]. In fact, as shown in Figure 5, aggregates of negatively charged LDPE particles with nanoscale features (~20 nm) were observed not only in the roots but also in the lower and upper hypocotyls, and even within the cotyledons. However, the signal intensity in the cotyledons was relatively weak compared to that in roots and appeared as localized fluorescence, making it less clearly distinguishable in the overall image. At this early growth stage, cotyledons represent the primary aerial tissues and therefore serve as an appropriate indicator of particle translocation from roots. This distribution visually demonstrates that LDPE particles with nanoscale features (~20 nm) particles taken up by the roots can migrate upward within the lettuce plant with the transpiration stream.

**Figure 7:**
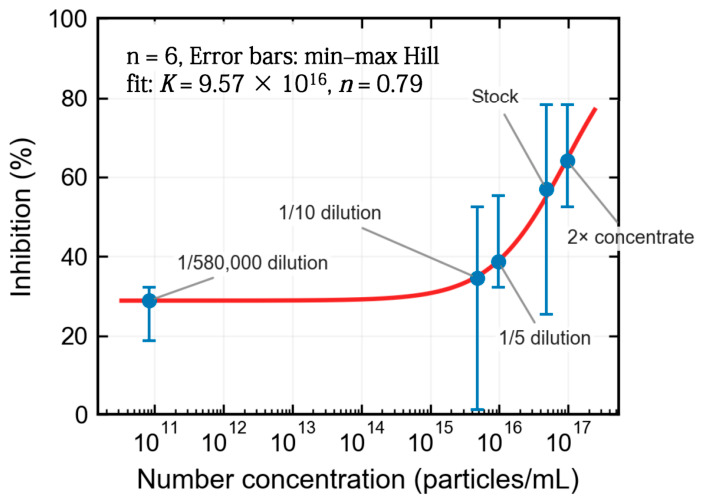
Relationship between particle number concentration (particles/mL) and growth inhibition (%) in hydroponically grown lettuce exposed to LDPE nanoparticles with nanoscale features (~20 nm). Blue symbols represent experimental data (error bars indicate min–max), and the red curve shows the constrained Hill model fit (K = 9.57 × 10^16^, *n* = 0.79). Labels indicate relative dilution and concentration levels with respect to the stock solution.

In recent years, it has become clear that micro- and nanoplastics in soil and water are taken up by plant roots under certain conditions, subsequently reaching the plant hypocotyl via the vascular cylinder, and then spreading to the cotyledons; as a result, the impact of these fine particles on plants is becoming a growing concern [33]. While quantitatively assessing the effects on plants is essential for elucidating the mechanisms of those effects, it is extremely difficult with current technology to quantitatively produce plastic particles—particularly degraded nanoplastics—and verify their inhibitory effects on plants by varying their number concentration; as a result, there have been virtually no such studies to date. Since we are able to prepare and degrade LDPE particles ourselves, we investigated the relationship between nanoparticle number concentration—a parameter that has rarely been examined in previous studies—and the inhibition of lettuce growth. Increasing particle number concentration resulted in a clear reduction in plant growth, particularly in terms of [root length/fresh weight/plant height]. This trend indicates a concentration-dependent inhibition effect, suggesting that higher exposure levels lead to stronger phytotoxic responses. This behavior is likely associated with increased interactions between particles and plant tissues, which may interfere with physiological processes such as water uptake and nutrient transport. These results demonstrate that particle number concentration is a critical factor governing plant responses to plastic particles, independent of mass-based metrics. These trends are illustrated in Figure 7. Concentration–response data were fitted using a constrained Hill equation, which has been widely applied to describe phytotoxicity and plant growth inhibition responses (as a sublethal response parameter) [14,15,34,35]. Similar shallow response patterns have been reported for nanoplastic-induced growth inhibition in lettuce [36,37]. In this study, the data were fitted using a constrained Hill equation, in which the maximum inhibition was fixed at 100% and the minimum inhibition was fixed at the observed value at the lowest concentration:

$$Inhibition\;\left(I\right)=I_{0}+\frac{1-I_{0}}{1+(K/C{)}^{n}}$$

*I*: Inhibition rate (0–100%). I_0_: Inhibition percentage at the lowest concentration point (fixed). *K* (*EC*_50_): Biologically unique half-maximal effective concentration. *n*: Hill coefficient. Although the *K* value (=9.57 × 10^16^) was obtained from the Hill model, in this paper we interpreted it not as a definitive toxicological threshold, but rather as a descriptive parameter characterizing the concentration–response relationship under the given constraints. In addition, the Hill coefficient represents the steepness and shape of the concentration–response curve and reflects how sensitively the response changes in response to changes in concentration. From a practical standpoint, n does not represent absolute potency itself, but rather determines how rapidly the effect increases near the inflection point (the midpoint of the response). In this study, the Hill coefficient was interpreted not as direct evidence of mechanistic synergy, but rather as a phenomenological parameter describing the steepness of the concentration–response curve. Therefore, we believe that the Hill coefficient (*n*) of 0.79, of less than 1, indicates a stepwise inhibition response, meaning that the inhibition of plant growth occurs across a wide range of concentrations rather than at a sharp threshold. Regarding the specific inhibition behavior, the *I* value remains nearly constant at 29% across a wide range of number concentrations, from 10^10^ to 10^15^. However, when the number concentration exceeds 10^16^, the *I* value increases exponentially, and a step-like change is observed, suggesting the presence of two distinct inhibition mechanisms.

### 3.3. Effects of Differences in Uptake Behavior and Shape of Nanoparticles in Different Parts of Lettuce on the Roots

**Figure 8:**
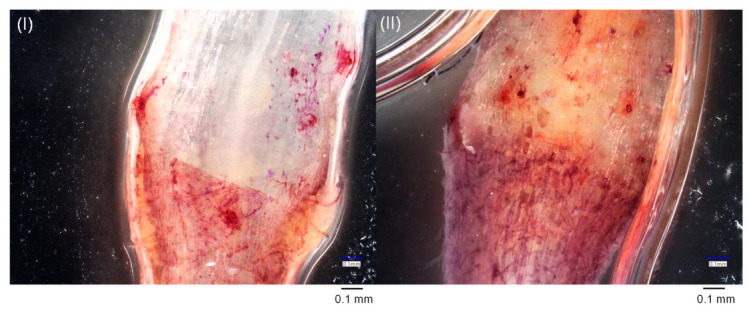
Laser microscopy cross-sectional images of the hypocotyl region of hydroponically grown lettuce exposed to iDye Poly Pink-stained (**I**) PS (2000 nm) microparticles (6.11 × 10^10^ particles/mL) and (**II**) LDPE particles with nanoscale features (~20 nm) nanoparticles (4.83 × 10^16^ particles/mL).

The two distinct inhibition mechanisms are inferred from the differences in staining patterns observed in the root (Figure 5a) and the lower hypocotyl (Figure 5b). While the presence of aggregated particles can be observed throughout the root, the photograph taken of the lower hypocotyl shows that the aggregates on the right side are relatively uniformly distributed, whereas those on the left side of the lower hypocotyl are localized. This region is the junction between the root and the lower hypocotyl, known as the collet, and serves as a transition zone [38]. Coordination between the primary meristems of the roots and stems involves spatial alignment between the systems, each of which has sections oriented in different directions [39]. This involves a transition from the typical root structure, which features an exogenous xylem, to a structure with an endogenous xylem typical of stems. In this transition zone (the hypocotyl), it appears that the NPs have become less mobile due to the structural changes in the xylem. It is possible that the diameter of the vascular bundles has narrowed or that the path has become curved, thereby limiting the size of particles that can pass through. By comparing particles with significantly different particle sizes, we can verify whether this size-selection effect exists. To verify the existence of a particle size-selection effect, we used stained PS (2000 nm), which consists of microsized particles, and compared its aggregation behavior near the embryonic axis with that of stained LDPE particles with nanoscale features (~20 nm). As shown in Figure 8, the boundary in the transition zone is more distinct for dyed PS (2000 nm) than for dyed LDPE particles with nanoscale features (~20 nm), clearly indicating that a size-selection effect is present in the collet. It should be noted that the “~20 nm” value corresponds to the peak size in the distribution, and larger particles are also present due to the heterogeneous nature of the system. This two-step inhibition behavior appears to be closely related to the size-selection mechanism operating in the collet. We believe that the I value of 29% is maintained across a wide range of number concentrations because the size-selection mechanism blocks the upward diffusion of NPs into the hypocotyl. However, since the particle size of LDPE NPs is small (approximately 20 nm), this blockage is not complete; when the number concentration exceeds 10^16^, the inhibitory effect due to NP aggregation in the hypocotyl and cotyledons becomes pronounced. In the low number concentration range, one of the causes of growth inhibition in hydroponically grown lettuce due to NP addition is NP aggregation occurring in the root region.

However, factors other than aggregation also contribute to the inhibition of root growth. The shapes of NPs and MPs are key determinants of plant interactions, influencing adhesion, uptake pathways, and physiological responses independently of size or polymer type [33,40,41,42,43,44]. Table 1 summarizes the inhibition rates of hydroponic lettuce when PS (600 nm) and PS (2000 nm) particles were added. All experiments were performed with *n* = 6 independent biological replicates. It should be noted, however, that while an apparent growth suppression was observed, the associated variability was large, resulting in substantial margins of error. Therefore, these values should be interpreted qualitatively rather than as precise quantitative inhibition thresholds in this context. For samples with extremely low suppression rates, PS has a higher specific gravity than water, so if the particles aggregate, they tend to settle. As a result, they cannot reach the roots, and the effect of particle addition cannot be realized. On the other hand, the high values that deviate significantly from the Hill plot of LDPE particles with nanoscale features (~20 nm) particles, despite the low number concentration, are due to differences in the inhibition mechanisms resulting from differences in particle shape.

**Figure 9:**
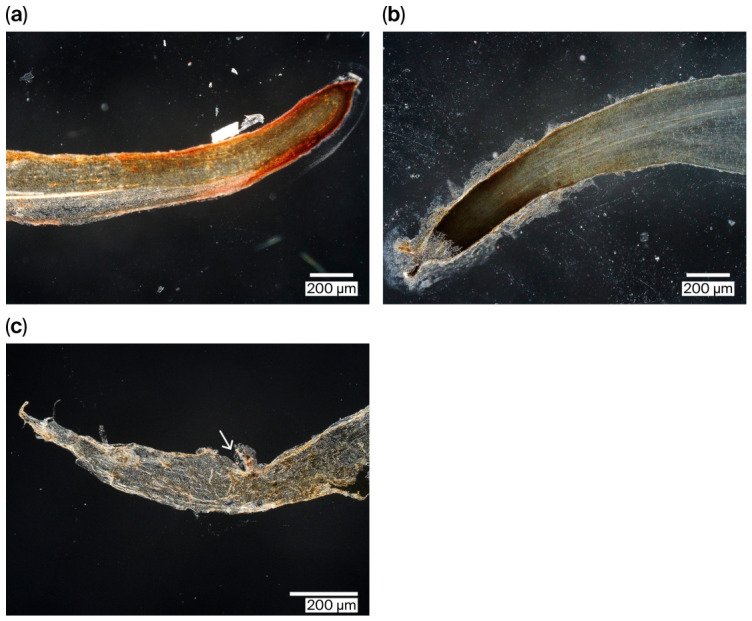
Laser microscopy images of roots of hydroponically grown lettuce: (**a**) control (pure water), (**b**) exposed to LDPE particles with nanoscale features (~20 nm) nanoparticles (4.83 × 10^16^ particles/mL), and (**c**) exposed to PS (600 nm) nanoparticles (1.32 × 10^12^ particles/mL). Arrows indicate the locations of PS particles and associated root surface damage.

Figure 9 shows laser microscope images of lettuce roots grown hydroponically with the addition of a control, LDPE particles with nanoscale features (~20 nm), and PS (600 nm) particles, respectively. These images illustrate the differences in particle uptake and localization within root tissues, reflecting size- and morphology-dependent interactions between particles and plant tissues. Figure 9 presents a conceptual representation of these behaviors based on the experimental observations. Therefore, the purpose of this figure is to summarize particle transport and interaction mechanisms rather than to provide detailed morphological characterization. No damage was observed on the roots of the control sample, and the epidermis was smooth. In contrast, the epidermis of the lettuce roots treated with LDPE particles with nanoscale features (~20 nm) particles became rough, indicating that even particles without sharp edges can damage the epidermis. The damage to the epidermis caused by PS (600 nm) particles with sharp edges is even more severe, making the entire root appear to be crumbling. The stronger growth inhibition observed for (600 nm) particles with sharp-edged morphology suggests that particle shape plays an important role in phytotoxicity. Unlike LDPE particles with nanoscale features (~20 nm), which are taken up and accumulated within plant tissues, the larger PS particles are likely to cause damage externally at the root surface, leading to growth inhibition. This damage to the root surface caused by the sharp edges of the particles is expected to be more pronounced when PS (2000 nm) particles are added, as these particles are inherently larger and less likely to penetrate the root. In fact, as shown in Table 1, the addition of PS (2000 nm) particles results in a higher inhibition rate compared to PS (600 nm) particles, despite their lower number concentration. These results indicate that, in some cases, the shape of the added particles can affect lettuce growth more significantly than the number concentration of the particles.

## 4. Conclusions

In this study, we systematically investigated the generation, characterization, and plant uptake behavior of environmentally relevant micro- and nanoplastics (MPs and NPs), with a particular focus on how particle size, shape, and number concentration influence growth inhibition in hydroponically grown lettuce. Significant oxidative degradation was required for the spontaneous formation of nanoscale particles during AOP treatment of LDPE. The resulting LDPE particles exhibited nanoscale features (~20 nm within a broad size distribution) and predominantly ellipsoidal morphology, which can be attributed to the selective delamination of cross-linked regions within the polymer matrix. In contrast, PS did not readily form nano- or microparticles under equivalent conditions but instead produced flake-like particles with sharp edges via mechanical fragmentation. These results indicate that particle size distribution and morphology are strongly governed by polymer type. Laser scanning microscopy revealed that LDPE particles were readily taken up by roots and translocated to hypocotyls and cotyledons, suggesting efficient internal transport facilitated by small particle size and surface charge. In contrast, larger PS particles exhibited limited translocation and primarily remained associated with root surfaces. FT-IR analysis further supported the accumulation of LDPE-derived components on root tissues. Quantitative analysis based on particle number concentration demonstrated a gradual, stepwise growth inhibition response for LDPE particles, which was well described by a constrained Hill model. This behavior suggests the presence of multiple inhibition mechanisms, including nanoparticle aggregation in root tissues at lower concentrations and increased particle transport and accumulation in aerial tissues at higher concentrations. These findings indicate that plant responses to nanoscale plastic particles occur over a broad concentration range rather than a single threshold. Comparative analysis with PS particles further demonstrated that particle morphology plays a critical role in phytotoxicity. Sharp-edged, flake-like PS particles caused pronounced physical damage to root tissues, resulting in significant growth inhibition even at relatively low particle concentrations. This observation highlights that particle-shape-induced physical interactions may, in some cases, exert a stronger influence on plant responses than particle size or number concentration alone. Overall, this study provides experimental evidence that plant responses to plastic particles are governed by the combined effects of particle size, shape, and number concentration. These results underscore the importance of using metrics beyond mass-based concentration and highlight the need to consider particle morphology and transport behavior in environmental risk assessments.

It should be noted that the present study is based on environmentally relevant heterogeneous particle systems, and particle size, shape, and polymer type were not independently controlled. In addition, high-resolution imaging techniques such as TEM were not employed for nanoscale structural characterization. Future studies should aim to decouple these factors and incorporate advanced analytical techniques, as well as investigations at later plant growth stages, to further elucidate the mechanisms underlying plant–nanoplastic interactions.

## Figures and Tables

**Table 1:** Number concentration and inhibition rate of PS particles with different sizes.

Sample	Number Conc. (Particles/mL)	Inhibit. Rate (%)
PS (600 nm)	1.3 × 10^12^	30 ± 35
PS (2000 nm)	6.1 × 10^10^	42 ± 43

## Data Availability

Data are contained within the article and Supplementary Materials.

## Supplementary Materials

The following supporting information can be downloaded at: https://www.mdpi.com/article/10.3390/polym18121436/s1, Figure S1: Particle size distribution of PS particles obtained after 15 days of AOP degradation: (A) Nano-sized PS [PS (600 nm)], (B) Micro-sized PS [PS (2000 nm)].
