# Supplementary material for: Size-, Shape-, and Number Concentration-Dependent Nanoplastics Accumulation and Growth Responses in Lettuce
Source: Polymers (Basel). 2026 Jun 9;18(12):1436. doi: 10.3390/polym18121436 (PMC13306561; doi:10.3390/polym18121436)
Supplement: Supplementary file 1 [file polymers-18-01436-s001.zip › polymers-4352358-supplementary.pdf]

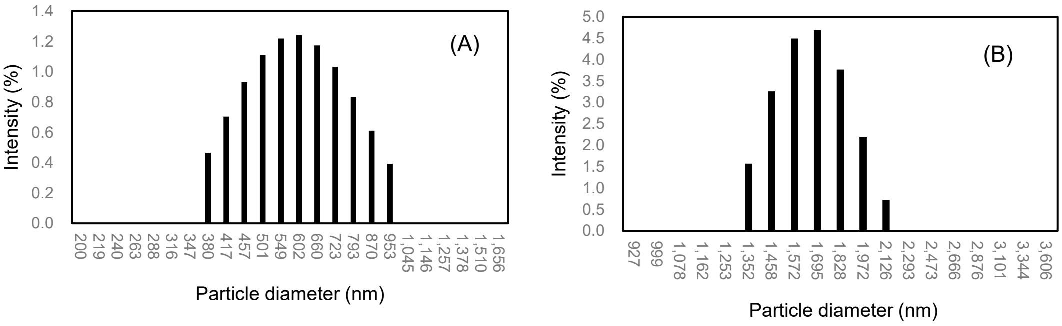

Figure S1. Particle size distribution of PS particles obtained after 15 days of AOP degradation: (A) Nano-sized PS [PS (600 nm)], (B) Micro- sized PS [PS (2000 nm)].
